# Regenerative Technologies: Future Grand Challenges and Emerging Strategies

**DOI:** 10.3389/fmedt.2020.603580

**Published:** 2020-11-06

**Authors:** Sarah H. Cartmell

**Affiliations:** Department of Materials, The University of Manchester, Manchester, United Kingdom

**Keywords:** therapeutics, regenerative medicine, tissue engineering, medical device, tools for healthcare

The United Nations 2019 World Population Prospects report has reported that by 2050, 1 in 6 people in the world will be over the age of 65, up from 1 in 11 in 2019 (1). As such, it is essential that we continue to develop new regenerative technologies that can address the associated growing and unmet clinical need of this aging and growing demograph. Consideration of the ease of translation of regenerative technology innovative products at all stages of research is important. This includes ease of manufacture scale up, regulatory aspects and also economic cost. The new aging population projections indicate that nine countries will make up more than half the projected growth of the global population between now and 2050. These include India, Nigeria, Pakistan, the Democratic Republic of the Congo, Ethiopia, the United Republic of Tanzania, Indonesia, Egypt, and the United States of America (in descending order). The 2019 United Nations report also states that circa 2027, India will overtake China as the world's country with the highest population (1). Therefore, an understanding of the health and economic needs of these particular areas is important to be considered when developing new products that will have a large global impact. Regenerative technology has the capability to address both niche areas of health but also can have a large global impact. The research area this journal covers is a broad theme covering innovative approaches that focuses on assisting the repair or replacement of damaged tissues and organs. Our speciality journal in regenerative technology aims to assist in dissemination and thus translation of these innovations in several areas.

## Regenerative Technologies for Therapeutics

Controlled release strategies are showing significant advances in regenerative technology for therapeutics. Recent advance examples include nanoclay technology for protein or drug delivery (2) or nanoparticle technology (3). Other researchers have presented the use of exosomes as a promising therapeutic approach (4). Recent research in minimally invasive surgical tool approaches also have shown advances in healthcare (5). One of our current biggest challenges in these areas include the fact that we are still needing to develop effective treatments for some major diseases such as Alzheimer's and some major cancers. From a research funding perspective, recently, governments are often preferring to fund preventative measures rather than new drug development and this therefore has a knock-on effect on drug innovation that could then subsequently be incorporated into controlled release strategies. However, we have made recent advances in the emerging technology of organ- and body-on-a-chip, which is promising as it opens up opportunities in drug discovery with respect to target identification, validation, target-based screening, and phenotype screening (6). A current challenge remains in developing improved *in vitro* dissolution or release testing for solid drug dosage forms, as current approaches often fail to predict *in vivo* performance. As such, the development of biorelevant dissolution tests that are mechanistically rather than empirically linked to *in vivo* pharmacokinetics would have impact on therapeutic technology (7).

## Clinical Applications of Regenerative Technologies

There are already several regenerative technologies being used in clinical practice, with Kim et al. stating in 2019 that currently “21 companies made an estimated $9 billion in sales of tissue engineering-related products in 2017. Based on previous reports and market trends, the field of tissue engineering is forecasted to continue to build revenue for the years to come” (8). There is also much emerging research being performed in this area as can be seen in recent publications that relate to examples such as stem cell technology (9), injectable biomaterials (10), imaging technologies (11), and 3D printing (12). Our biggest challenges in this area lies not really with the innovation, of which there is much research, but in the translation of the innovation. This requires our researchers to have an understanding of the road map of the product translation and build this consideration into the research planning at early stages. Patient and clinical input at every stage of the research to help guide the innovation development will assist in moving the product through to clinic. It also requires our researchers to network effectively—not just with other disciplines but with industry and regulatory experts in order to ensure smooth clinical translation. As such publication of clinical trials and preclinical assessment of developing products is important in this field to help guide others not only in their understanding of the new product but also how to plan such trials to develop other new tools.

## Quantitative and Qualitative Engineering Tools for Understanding Cell/Tissue Behavior

Exciting new research in developing new tools is emerging with recent examples including novel microarray approaches (13), extracellular analog technology to measure cell—extracellular matrix dynamics (14) and electrical stimulation (15). Our challenges in this area include improving resolution and accuracy of imaging technology and being able to assess larger samples of composite structures. Such composite structures may consist of both hard and soft matrices, with most technologies designed to assess either hard or soft matrices and find it difficult to assess a combination of the two. Assessment of cells encapsulated in matrices that interfere with currently used techniques (such as poor uptake of fluorescent dye in cells encapsulated in a 3D matrix due to low permeability or over expression of a fluorescent dye due to the matrix also becoming labeled with the dye as well as the cells) is also an issue in some laboratories. Our journal has a focus on improving translation and as such, we have a need for tools to improve the scale up of both manufacture and assessment of our new regenerative technology products. Tools, for example, to improve the speed of manufacture of electrospun scaffold blends to produce enough material to be tested for preclinical testing, before a decision can be made to move to industrial scale production, would not only reduce the time taken for translation, but also allow the work not to be halted at this point due to time and economic constraints.

## Bioprinting Technologies for Construction of Multi-Cellular/Multi-Material Tissues

Bioprinting in both 2D and 3D has recently gained enormous attention due to its flexibility in printing multiple materials / cell types at once but also the creation of complex structures whilst incorporating important growth factors etc. As Zhang and Zhang state in 2015, “A major advantage of this technology is its ability for simultaneously 3D printing various cell types in defined spatial locations, which makes this technology applicable to regenerative medicine to meet the need for suitable for transplantation suitable organs and tissues” (16). Our biggest challenge in this area now is still to create the technology to print 3D structures that closely resemble organs or tissues. Development in the mechanical integrity of the currently printed structures as well as improving printing speed and accuracy. Further research into understanding the effects of printing on the cells and materials themselves is also still needed.

## Novel Therapeutic Tissue Repair Devices

Tissue engineered constructs are at various stages of development for a wide variety of disease treatments and damaged tissue replacement. Examples include diabetes innovation (17), osteoarthritis approaches (18), and cardiovascular tissue replacement (19). Our challenges in this area include scaling up the manufacture size and number of these products (bioreactor technology development necessary in this area), increasing the speed of manufacture (off the shelf vs. a 6 week patient wait before implantation) and improving the functionality of the end product. This could mean improving the mechanical integrity of the construct for example if in a load bearing role or perhaps improving the transparency of the product or electrical conductivity. Ensuring quality assurance and control of manufacture which will include reproductivity and reliability of these constructs is important for clinical translation. Understanding how the construct performs when created with aged human patient cells vs. a murine cell line in a lab testing is also important to consider. However, with the plethora of emerging technology in areas such as fiber manufacture, bioprinting, nanomaterials and cell biology, we are at the edge of exciting technologies to be created in this area.

## Advances in Approaches in Regenerative Technology Policy and Regulations

As mentioned at the beginning of this article, the demograph of target patient profile is important to be considered. Therefore, researchers that wish to translate their research in this very applied area, should always consider the regulatory implications of their target health market. These regulatory considerations are constantly under review and changing as can be seen with recent developments in EU regulatory policy and with future changes expected as a result of Brexit within Northern Ireland and Great Britain which will need to be considered for United Kingdom healthcare regenerative medical product translation. Schuh et al. have recently discussed the differences between regenerative medical products and medical devices and stated that “regenerative products may have medical device-based scaffolding and may be treated as biologics, reflecting the cell, and tissue components. This compilation of international standards and guidelines provides toxicologic pathologists, toxicologists, bioengineers, and allied professionals with an overview of and source for important regulatory documents that may apply to the nonclinical development of their products” (20). Testing appropriate to these standards can easily be performed in parallel, rather than in sequence to research innovation, which could significantly reduce the time taken to translate to the clinic. An awareness of the regulatory procedure appropriate for the target product market should also be considered as a complete regulatory dossier is easier to compile during the process than at the end. Our new technologies may also fall into a category that may not be easily defined by current regulations. As such, dialogue and influence on regulatory policy is important to ensure fit for purpose testing is in place. Research into this area to improve preclinical testing is needed. Examples of this could include human cell organ on a chip testing prior to first in man clinical trials to assess human cell phenotype response to a new technology.

## Conclusions

To conclude, regenerative technology is an exciting, fast moving and much needed research area. With current interdisciplinary approaches and researchers informed not only in the science and engineering aspects but also with an understanding of the regulatory and clinical/industry road map needed for their products, we have the potential to make a huge impact in patient quality of life and health economics. It is important as we perform this work that we also consider the sustainability aspects of our work and include reflection on responsible research in our innovation.

## Author Contributions

The author confirms being the sole contributor of this work and has approved it for publication.

## Conflict of Interest

The author declares that the research was conducted in the absence of any commercial or financial relationships that could be construed as a potential conflict of interest.
